# Internet and Gaming Addiction: A Systematic Literature Review of Neuroimaging Studies

**DOI:** 10.3390/brainsci2030347

**Published:** 2012-09-05

**Authors:** Daria J. Kuss, Mark D. Griffiths

**Affiliations:** International Gaming Research Unit, Nottingham Trent University, Nottingham NG1 4BU, UK; Email: mark.griffiths@ntu.ac.uk

**Keywords:** Internet addiction, gaming addiction, neuroimaging, literature review

## Abstract

In the past decade, research has accumulated suggesting that excessive Internet use can lead to the development of a behavioral addiction. Internet addiction has been considered as a serious threat to mental health and the excessive use of the Internet has been linked to a variety of negative psychosocial consequences. The aim of this review is to identify all empirical studies to date that used neuroimaging techniques to shed light upon the emerging mental health problem of Internet and gaming addiction from a neuroscientific perspective. Neuroimaging studies offer an advantage over traditional survey and behavioral research because with this method, it is possible to distinguish particular brain areas that are involved in the development and maintenance of addiction. A systematic literature search was conducted, identifying 18 studies. These studies provide compelling evidence for the similarities between different types of addictions, notably substance-related addictions and Internet and gaming addiction, on a variety of levels. On the molecular level, Internet addiction is characterized by an overall reward deficiency that entails decreased dopaminergic activity. On the level of neural circuitry, Internet and gaming addiction led to neuroadaptation and structural changes that occur as a consequence of prolonged increased activity in brain areas associated with addiction. On a behavioral level, Internet and gaming addicts appear to be constricted with regards to their cognitive functioning in various domains. The paper shows that understanding the neuronal correlates associated with the development of Internet and gaming addiction will promote future research and will pave the way for the development of addiction treatment approaches.

## 1. Introduction

In the past decade, research has accumulated suggesting that excessive Internet use can lead to the development of a behavioral addiction (e.g., [1,2,3,4]). Clinical evidence suggests that Internet addicts experience a number of biopsychosocial symptoms and consequences [5]. These include symptoms traditionally associated with substance-related addictions, namely salience, mood modification, tolerance, withdrawal symptoms, conflict, and relapse [6]. Internet addiction comprises a heterogeneous spectrum of Internet activities with a potential illness value, such as gaming, shopping, gambling, or social networking. Gaming represents a part of the postulated construct of Internet addiction, and gaming addiction appears to be the most widely studied specific form of Internet addiction to date [7]. Mental health professionals’ and researchers’ extensive proposals to include Internet addiction as mental disorder in the forthcoming fifth edition of the Diagnostic and Statistical Manual of Mental Disorders (DSM-V) will come to fruition as the American Psychiatric Association accepted to include *Internet use disorder* as mental health problem worthy of further scientific investigation [8]. 

The excessive use of the Internet has been linked to a variety of negative psychosocial consequences. These include mental disorders such as somatization, obsessive-compulsive and other anxiety disorders, depression [9], and dissociation [10], as well as personality traits and pathology, such as introversion and psychoticism [11]. Prevalence estimates range from 2% [12] to 15% [13], depending on the respective sociocultural context, sample, and assessment criteria utilized. Internet addiction has been considered as serious threat to mental health in Asian countries with extensive broadband usage, particularly South Korea and China [14].

### 1.1. The Rise of Neuroimaging

In accordance with Cartesian dualism, the French philosopher Descartes advocated the view that the mind is an entity that is separate from the body [15]. However, the cognitive neurosciences have proved him wrong and reconcile the physical entity of the body with the rather elusive entity of the mind [16]. Modern neuroimaging techniques link cognitive processes (*i.e.*, Descartes’ *thinking mind*) to actual behavior (*i.e.*, Descartes’ *moving body*) by measuring and picturing brain structure and activity. Altered activity in brain areas associated with reward, motivation, memory, and cognitive control has been associated with addiction [17]. 

Research has addressed the neural correlates of drug addiction development via classical and operant conditioning [18,19]. It has been found that during the initial stages of the voluntary and controlled usage of a substance, the decision to use the drug is made by specific brain regions, namely the prefrontal cortex (PFC) and ventral striatum (VS). As habituation to use and compulsion develops, brain activity changes in that the dorsal regions of the striatum (DS) become increasingly activated via dopaminergic innervation (*i.e.*, dopamine release) [20]. Long term drug use leads to changes in the brain dopaminergic pathways (specifically the anterior cingulate (AC), orbitofrontal cortex (OFC), and the nucleus accumbens (NAc) which may lead to a reduction of sensitivity to biological rewards and it decreases the individual’s control over seeking and eventually taking drugs. [21,22]. On a molecular level, the long-term depression (LTD; *i.e.*, the reduction) of synaptic activity has been linked to the adaptation of the brain as a result of substance-related addictions [23]. Drug addicts become sensitized to the drug because in the course of prolonged intake, the synaptic strength in the ventral tegmental area increases, and so does the LTD of glutamate in the nucleus accumbens, which will result in craving [24].

At the same time, the brain (*i.e.*, NAc, OFC, DLPFC) becomes increasingly responsive to drug cues (e.g., availability, particular context) via craving [21,25]. Craving for drug use involves a complex interaction between a variety of brain regions. Activity in the nucleus accumbens following recurrent drug intake leads to learning associations between drug cues and the reinforcing effects of the drug [26]. In addition, the orbitofrontal cortex, important for the motivation to engage in behaviors, the amygdala (AMG) and the hippocampus (Hipp), as main brain regions associated with memory functions, play a role in intoxication and craving for a substance [17].

Natural rewards, such as food, praise, and/or success gradually lose their hedonic valence. Due to habituation to rewarding behaviors and intake of drugs, a characteristic addiction symptom develops (*i.e.*, tolerance). Increasing amounts of the substance or increasing engagement in the respective behaviors are needed in order to produce the desired effect. As a result, the reward system becomes deficient. This leads to the activation of the antireward system that decreases the addict’s capacity for experiencing biological reinforcers as pleasurable. Instead, he requires stronger reinforcers, *i.e.*, their drug or behavior of choice, in larger amounts (*i.e.*, tolerance develops) to experience reward [27]. In addition, the lack of dopamine in the mesocorticolimbic pathways during abstinence explains characteristic withdrawal symptoms. These will be countered with renewed drug intake [17]. Relapse and the development of a vicious behavioral cycle are the result [28]. Prolonged drug intake and/or engagement in a rewarding behavior leads to changes in the brain, including dysfunctions in prefrontal regions, such as the OFC and the cingulate gyrus (CG) [17,29].

Research indicates that brain activity alterations commonly associated with substance-related addictions occur following the compulsive engagement in behaviors, such as pathological gambling [30]. In line with this, it is conjectured that similar mechanisms and changes are involved in Internet and gaming addiction. The aim of this review is therefore to identify all peer-reviewed empirical studies to date that used neuroimaging techniques to shed light upon the emerging mental health problem of Internet and gaming addiction from a neuroscientific perspective. Neuroimaging broadly includes a number of distinct techniques. These are Electroencephalogram (EEG), Positron Emission Tomography (PET), SPECT Single Photon Emission Computed Tomography (SPECT), functional Magnetic Resonance Imaging (fMRI), and structural magnetic resonance imaging (sMRI), such as Voxel-based Morphometry (VBM), and Diffusion-Tensor Imaging (DTI). These are briefly explained in turn before examining the studies that have utilized these techniques for studies on Internet and gaming addiction.

### 1.2. Types of Neuroimaging Used to Study Addictive Brain Activity

*Electroencephalogram (EEG):* With an EEG, neural activity in the cerebral cortex can be measured. A number of electrodes are fixed to specific areas (*i.e.*, anterior, posterior, left and right) of the participant’s head. These electrodes measure voltage fluctuations (*i.e.*, current flow) between pairs of electrodes that are produced by the excitation of neuronal synapses [31]. With event-related potentials (ERPs), the relationships between the brain and behavior can be measured via an electrophysiological neuronal response to a stimulus [32].

*Positron Emission Tomography (PET):* PET is a neuroimaging method that allows for the study of brain function on a molecular level. In PET studies, metabolic activity in the brain is measured via photons from positron emissions (*i.e.*, positively charged electrons). The subjected is injected with a radioactive 2-deoxyglucose (2-DG) solution that is taken up by active neurons in the brain. The amounts of 2-DG in neurons and positron emissions are used to quantify metabolic activity in the brain. Thus, neuronal activity can be mapped during the performance of a particular task. Individual neurotransmitters can be distinguished with PET, which makes the latter advantageous over MRI techniques. It can measure activity distribution in detail. Limitations to PET include relatively low spatial resolution, time needed to obtain a scan, as well as potential radiation risk [33].

*Single Photon Emission Computed Tomography (SPECT):* SPECT is a subform of PET. Similar to PET, a radioactive substance (a “tracer”) is injected into the blood stream that rapidly travels to the brain. The stronger the metabolic activity in specific brain regions, the stronger the enrichment of gamma rays. The emitted radiation is measured in accordance with brain layers, and metabolic activity is imaged using computerized techniques. Unlike PET, SPECT allows for counting individual photons, however, its resolution is poorer because with SPECT, resolution depends on the proximity of the gamma camera that measures neuronal radioactivity [34].

*Functional Magnetic Resonance Imaging (fMRI):* With fMRI, changes in the levels of blood oxygen in the brain are measured that are indicative of neuronal activity. Specifically, the ratio of oxyhemoglobin (*i.e.*, hemoglobin that contains oxygen in the blood) to deoxyhemoglobin (*i.e.*, hemoglobin that has released oxygen) in the brain is assessed because blood flow in “active” brain areas increases to transport more glucose, also bringing in more oxygenated hemoglobin molecules. The assessment of this metabolic activity in the brain allows for finer and more detailed imaging of the brain relative to structural MRI. In addition to this, the advantages of fMRI include speed of brain imaging, spatial resolution, and absence of potential health risk relative to PET scans [35].

*Structural Magnetic Resonance Imaging (sMRI):* sMRI uses a variety of techniques to image brain morphology [36]. One such technique is *Voxel-Based Morphometry (VBM)*. VBM is used to compare the volume of brain areas and the density of gray and white matter [37]. Another sMRI technique is *Diffusion-Tensor Imaging (DTI)*. DTI is a method used for picturing white matter. It assesses the diffusion of water molecules in the brain which helps to identify interconnected brain structures by using fractional anisotropy (FA). This measure is an indicator of fiber density, axonal diameter, and myelination in white matter [38]. 

## 2. Method

A comprehensive literature search was conducted using the database *Web of Knowledge*. The following search terms (and their derivatives) were entered with regards to Internet use: “addiction”, “excess”, “problem”, and “compulsion”. Moreover, additional studies were identified from supplementary sources, such as *Google Scholar*, and these were added in order to generate a more inclusive literature review. Studies were selected in accordance with the following inclusion criteria. Studies had to (i) assess Internet or online gaming addiction or direct effects of gaming on neurological functioning, (ii) use neuroimaging techniques, (iii) be published in a peer-reviewed journal, and (iv) be available as full text in English language. No time period was specified for the literature search because neuroimaging techniques are relatively new, so that the studies were expected to be recent (*i.e.*, almost all having been published between 2000 and 2012). 

## 3. Results

A total of 18 studies were identified that fulfilled the inclusion criteria. Of those, the method of data acquisition was fMRI in eight studies [39,40,41,42,43,44,45,46] and sMRI in two studies [47,48], two studies used PET scans [49,50], one of which combined it with an MRI [49], one used SPECT [51], and six studies utilized EEG [52,53,54,55,56,57]. It should also be noted that two of these were actually the same study with one published as a letter [53] and one published as a full paper [54]. One study [57] met all the criteria but was excluded because the diagnosis details of Internet addiction were insufficient to make valid conclusions. Furthermore, two studies did not directly assess Internet and gaming addiction [43,50], but assessed the direct effects of gaming on neurological activity using an experimental paradigm, and were therefore retained in the review. Detailed information on the included studies are presented in Table 1.

### 3.1. fMRI Studies

Hoeft *et al*. [43] investigated gender differences in the mesocorticolimbic system during computer-game play among 22 healthy students (age range = 19–23 years; 11 females). All participants underwent fMRI (3.0-T Signa scanner (General Electric, Milwaukee, WI, USA), completed the Symptom Checklist 90-R [58], and the NEO-Personality Inventory-R [59]. FMRI was carried out during 40 blocks of either a 24-s ball game with the goal being to gain space or a similar control condition that did not include a specific game goal (as based on its structural makeup). Results indicated that there was an activation of neural circuitries that are involved in reward and addiction in the experimental condition (*i.e.*, insula, NAc, DLPFC, and OFC). Consequently, the presence of an actual game goal (a characteristic of most conventional online games that are rule-based rather than pure role-playing games), modified brain activity via behavior. Here, a clear cause and effect relationship is evident, which adds strength to the findings.

Results also showed that male participants had a larger activation (in rNAc, blOFC, rAMG) and functional connectivity (lNAc, rAMG) in the mesocorticolimbic reward system when compared to females. The results furthermore indicated that playing the game activated the right insula (rI; signals autonomic arousal), right dorso-lateral PFC (maximize reward or change behavior), bilateral premotor cortices (blPMC; preparation for reward) and the precuneus, lNAc, and the rOFC (areas involved in visual processing, visuo-spatial attention, motor function, and sensori-motor transformation) compared to the resting state [43]. The insula has been implicated in conscious craving for addictive substances by implicating decision-making processes involving risk and reward. Insula dysfunction may explain neurological activities indicative of relapse [60]. Due to its experimental nature, this study was able to provide insight into idiosyncratic brain activation as a consequence of gaming in a healthy (*i.e.*, non-addicted) population.

**Table 1:** Included studies.

Study (Year)	Main Aims	Sample [Design/Method]	Internet addiction diagnosis	Main Results
Dong, Huang & Du [39]	Examined reward and punishment processing in Internet addicts versus healthy controls	14 male Internet addicts	Internet Addiction Test [61]; Chinese Internet Addiction Test [62,63]	Internet addiction associated with increased activation in orbitofrontal cortex in gain trials, decreased anterior cingulate activation in loss trials compared to normal controls; Enhanced reward sensitivity and decreased loss sensitivity than normal controls
		13 healthy males		
		[Reality-simulated fMRI quasi-experimental guessing task for money gain or loss situation using playing cards]		
Dong, Zhou & Zhao [52]	Investigated executive control ability of Internet addicts	17 male Internet addicts	Internet Addiction Test [64]	Internet addicts had longer reaction time and more response errors in incongruent conditions than controls; reduced medial frontal negativity (MFN) deflection in incongruent conditions than controls
		17 male healthy university students		
		[Measured event-related potentials (ERP) via electroencephalogram (EEG) during a quasi-experimental color-word Stroop task]		
Dong, Lu, Zhou & Zhao [53]	Investigated neurological response inhibition in Internet addicts	12 male Internet addicts	Internet Addiction Test [65]	Internet addicts had (i) lower NoGo-N2 amplitudes (represent response inhibition-conflict monitoring), higher NoGo-P3 amplitudes (inhibitory processes—response evaluation), (ii) longer NoGo-P3 peak latency than controls, and (iii) less efficient information processing and lower impulse control
		12 male healthy control university students		
		[Quasi-experimental EEG study: Recordings of event-related brain potentials (ERPs) via EEG during a quasi-experimental go/NoGo task]		
Ge, Ge, Xu, Zhang, Zhao & Kong [66]	Investigated association between P300 component and Internet addiction disorder	38 Internet addiction patients (21 males)	Internet Addiction Test [64]	Study found similar results for Internet addicts as compared to other substance-related addicts; Cognitive dysfunctions associated with Internet addiction can be improved Internet addicts had longer P300 latencies relative to controls
		48 healthy college student controls (25 males)		
		[Quasi-experimental EEG study; P300 ERP measured using standard auditory oddball task using American Nicolet BRAVO instrument]		
Han, Lyoo & Renshaw [40]	Compared regional gray matter volumes in patients with online game addiction (POGA) and professional gamers (PGs)	20 patients with online game addiction	Young’s Internet Addiction Scale [67]	POGA had higher impulsiveness, perseverative errors, volume in left thalamus gray matter, decreased gray matter volume in inferior temporal gyri, right middle occipital gyrus, left inferior occipital gyrus relative to HC;PGs had increased gray matter volume in left cingulate gyrus, decreased in left middle occipital gyrus and right inferior temporal gyrus relative to HC, and increased in left cingulate gyrus and decreased left thalamus gray relative to POGA
		17 pro-gamers		
		18 healthy male controls		
		[fMRI study with voxel-wise comparisons of gray matter volume]		
Han, Hwang & Renshaw [41]	Tested effects of bupropion sustained release treatment on brain activity for online video game addicts	11 male Internet video game addicts	Young’s Internet Addiction Scale [67]; Craving for Internet Video Game Play Scale	During exposure to game cues, IGA had more brain activation in left occipital lobe cuneus, left dorsolateral prefrontal cortex, left parahippocampal gyrus relative to H; After treatment, craving, play time, and cue-induced brain activity decreased in IAG
		8 healthy male controls		
		[Quasi-experimental fMRI study at baseline and after six weeks of treatment]		
Han, Kim, Lee, Min & Renshaw [42]	Assessed differences in brain activity between baseline and video game play	21 university students (14 males)	Young’s Internet Addiction Scale [67]; Craving for Internet Video Game Play Scale	Brain activity in anterior cingulate and orbitofrontal cortex increased in excessive Internet game playing group (EIGP) following exposure to Internet video game cues relative to general players (GP); Increased craving for Internet video games correlated with increased activity in anterior cingulate for all participants
		[Quasi-experimental fMRI study at baseline and after six weeks of videogame play]		
Hoeft, Watson, Kesler, Bettin-ger & Reiss [43]	Investigated gender differences in mesocorti-colimbic system during computer-game play	22 healthy students (11 males)	Addiction not assessed via self-report	Activation of neural circuitries involved in reward and addiction ( *i.e.*, nucleus accumbens, amygdala, dorso-lateral prefrontal cortex, insular cortex, and orbitofrontal cortex); Males had a larger activation (in right nucleus accumbens, bilateral orbitofrontal cortex, right amygdala) and functional connectivity (left nucleus accumbens and right amygdala) in mesocorticolimbic reward system relative to females
		[Experimental fMRI study performed with 3.0-T Signa scanner (General Electric, Milwaukee, WI, USA) 40 blocks of either 24 s ball game or control condition]		
Hou, Jia, Hu, Fan, Sun, Sun & Zhang [51]	Examined reward circuitry dopamine transporter levels in Internet addicts compared to controls	5 male Internet addicts	Young’s Internet Addiction Diagnostic Questionnaire [64]; Internet addictive Disorder Diagnostic Criteria [68]	Reduced dopamine transporters indicate addiction: similar neurobiological abnormalities with other behavioural addictions; Striatal dopamine transporter (DAT) levels decreased in Internet addicts (necessary for regulation of striatal dopamine levels) and volume, weight, and uptake ratio of the corpus striatum were reduced; Dopamine levels similar in people with substance addiction
		9 healthy age-matched male controls		
		[SPECT study: 99mTc-TRODAT-1 single photon emission computed tomography (SPECT) brain scans using Siemens Diacam/e.cam/icon double detector]		
Kim, Baik, Park, Kim, Choi & Kim [49]	Tested if Internet addiction is associated with reduced levels of dopaminergic receptor availability in the striatum	5 male Internet addicts	Internet Addiction Test [69]; Internet Addictive Disorder Diagnostic Criteria [68]	Internet addicts had reduced dopamine D2 receptor availability in striatum ( *i.e.*, bilateral dorsal caudate, right putamen);Negative correlation of dopamine receptor availability with Internet addiction severity;Internet addiction found to be related to neurobiological abnormalities in the dopaminergic system as found in substance-related addictions
		7 male controls		
		[PET study: Radiolabeled ligand [^11^C]raclopride and positron emission tomorgraphy via ECAT EXACT scanner used to test dopamine D2 receptor binding potential; fMRI using General Electric Signa version 1.5T MRI scanner; Method for assessing D2 receptor availability: regions of interest (ROI) analysis in ventral striatum, dorsal caudate, dorsal putamen]		
Ko, Liu, Hsiao, Yen, Yang, Lin, Yen & Chen [44]	Identified neural substrates of online gaming addiction by assessing brain areas involved in urge	10 male online gaming addicts	Chen Internet Addiction Scale (CIAS) [71]	Dissimilar brain activation in gaming addicts: right orbitofrontal cortex, right nucleus accumbens, bilateral anterior cingulate, medial frontal cortex, right dorsolateral prefrontal cortex, right caudate nucleus and this correlated with gaming urge and recalling of gaming experience; Cue induced craving common in substance dependence: similar biological basis of different addictions including online gaming addiction
		[Quasi-experimental fMRI study: Presentation of gaming-related and paired mosaic pictures during fMRI scanning (3T MRscanner); Contrasts in BOLD signals in both conditions analysed; Cue reactivity paradigm] [70]		
Koepp, Gunn, Law-rence, Cunning-ham, Dagher, Jones, Brooks, Bench & Grasby [50]	Provided evidence for striatal dopamine release during a video game play	8 males	Addiction not assessed via self-report	Reduction of binding of raclopride to dopamine receptors in striatum during video game play relative to baseline; Correlation between performance level and reduced binding potential in all striatal regions; First study to show that dopamine is released during particular behaviours;Ventral and dorsal striata associated with goal-directed behaviour
		[Experimental PET study 953B-Siemens/CTIPET camera; Positron emission tomography (PET) during video game play and under resting condition; Region-of-interest (ROI) analysis;Extracellular dopamine levels measured via differences in [^11^C]RAC-binding potential to dopamine D_2_ receptors in ventral and dorsal striata]		
Lin, Zhou, Du, Qin, Zhao, Xu & Lei [48]	Investigated white matter integrity in adolescent Internet addicts	17 Internet addicts (14 males)	Modified Young’s Internet Addiction Test [72]	Internet addicts had lower FA throughout the brain (orbito-frontal white matter corpus callosum, cingulum, inferior fronto-occipital fasciculus, corona radiation, internal and external capsules);Negative correlations between FA in left genu of corpus callosum and emotional disorders, and FA in left external capsule and Internet addiction; Similarities in brain structures between Internet and substance addicts
		16 healthy controls (14 males)		
		[Whole brain voxel-wise analysis of fractional anisotropy (FA) by tract-based spatial statistics (TBSS) and volume of interest analysis were performed using diffusion tensor imaging (DTI) via a 3.0-Tesla Phillips Achieva medical scanner]		
Littel, Luijten, van den Berg, van Rooij, Kee-mink & Franken [56]	Investigated error-processing and response inhibition in excessive gamers	25 excessive gamers (23 males)	Videogame Addiction Test (VAT) [53]	Similarities with substance dependence and impulse control disorders regarding poor inhibition, high impulsivity in excessive gamers; Excessive gamers: reduced fronto-central ERN amplitudes following incorrect trials in comparison to correct trials leading to poor error-processing
		27 controls (10 males)		
		[Electroencephalography (EEG): Go/NoGo paradigm using EEG and ERP recordings]		
Liu, Gao, Osunde, Li, Zhou, Zheng & Li [45]	Applied regional homogeneity method to analyse encephalic functional characteristic of Internet addicts in resting state	19 college students with Internet addiction (11 males and 8 females)	Modified Diagnostic Questionnaire for Internet Addiction [72]	Internet addicts suffer from functional brain changes leading to abnormalities in regional homogeneity in Internet addicts relative to controls; Internet addicts had increased brain regions in ReHo in resting state (cerebellum, brainstem, right cingulate gyrus, bilateral parahippocampus, right frontal lobe, left superior frontal gyrus, right inferior temporal gyrus, left superior temporal gyrus and middle temporal gyrus)
		19 controls (gender matched)		
		[fMRI study: Functional magnetic resonance image using 3.0T Siemens Tesla Trio Tim scanner; Assessed resting state fMRI; Regional homogeneity (ReHo) indicates temporal homogeneity of regional BOLD signal rather than its density]		
Yuan, Qin, Wang, Zeng, Zhao, Yang, Liu, Liu, Sun, von Deneen, Gong, Liu & Tian [46]	Investigated effects of Internet addiction on the microstructural integrity of major neuronal fiber pathways and microstructural changes with duration of Internet addiction	18 students with Internet addiction (12 males)	Modified Diagnostic Questionnaire for Internet Addiction [72]	Increased FA of left posterior limb of internal capsule (PLIC) and reduced FA in white matter in right parahippocampal gyrus (PHG); Correlation between gray matter volumes in DLPFC, rACC, SMA, and white matter FA changes of PLIC with Internet addiction length; Internet addiction results in changes in brain structure
		18 control subjects (gender matched)		
		[fMRI study: Optimised voxel-based morphometry (VBM) technique. Analysed white matter fractional anisotropy (FA) changes by using diffusion tensor imaging (DTI) to associate brain structural changes to Internet addiction length]		
Zhou, Lin, Du, Qin, Zhao, Xu & Lei [47]	Investigated brain gray matter density (GMD) changes in adolescents with Internet addiction using voxel-based morphometry (VBM) analysis on high-resolution T1-weighted structural magnetic resonance images	18 adolescents with Internet addiction (2 females)	Modified Diagnostic Questionnaire for Internet Addiction [72]	Structural brain changes in adolescents with Internet addiction; Internet addicts had lower GMD in left anterior cingulate cortex (necessary for motor control, cognition, motivation), left posterior cingulate cortex (self-reference), left insula (specifically related to craving and motivation)
		15 healthy controls (2 females)		
		[MRI study: Used high-resolution T1-weighted MRIs performed on a 3T MR scanner (3T Achieva Philips), scanned MPRAGE pulse sequences for gray and white matter contrasts; VBM analysis to compare GMD between groups]		

Ko *et al*. [44] attempted to identify the neural substrates of online gaming addiction by assessing brain areas involved in urge to engage in online games among ten male online gaming addicts (playing *World of Warcraft* for more than 30 h a week) compared to ten male controls (whose online use was less than two hours a day). All participants completed the Diagnostic Criteria for Internet Addiction for College Students (DCIA-C; [74]), the Mini-International Neuropsychiatric Interview [75], the Chen Internet Addiction Scale (CIAS) [71], the Alcohol Use Disorder Identification Test (AUDIT) [76], and the Fagerstrom Test for Nicotine Dependence (FTND) [77]. The authors presented gaming-related and paired mosaic pictures during fMRI scanning (3T MRscanner), and contrasts in BOLD signals in both conditions were analyzed using a cue reactivity paradigm [25]. The results indicated cue induced craving that is common among those with substance dependence. There was a dissimilar brain activation among gaming addicts following the presentation of game relevant cues as compared to controls and compared to the presentation of mosaic pictures, including the rOFC, rNAc, blAC, mFC, rDLPFC, and the right caudate nucleus (rCN). This activation correlated with gaming urge and a recalling of gaming experience. It was argued that there is a similar biological basis of different addictions including online gaming addiction. The quasi-experimental nature of this study that artificially induced craving in an experimental and controlled setting allowed the authors to make conclusions as based on group differences, and thus linking online gaming addiction status to the activation of brain areas associated with symptoms of more traditional (*i.e.*, substance-related) addictions. 

Han *et al*. [42] assessed the differences in brain activity before and during video game play in university students playing over a seven-week period. All participants completed the Beck Depression Inventory [78], the Internet Addiction Scale [67], and a 7-point visual analogue scale (VAS) to assess craving for Internet video game play. The sample comprised 21 university students (14 male; mean age = 24.1 years, SD = 2.6; computer use = 3.6, SD = 1.6 h a day; mean IAS score = 38.6, SD = 8.3). These were further divided into two groups: the excessive Internet gaming group (who played Internet video games for more than 60 min a day over a 42-day period; *n* = 6), and general player group (who played less than 60 min a day over the same period; *n* = 15). The authors used 3T blood oxygen level dependent fMRI (using Philips Achieva 3.0 Tesla TX scanner) and reported that brain activity in the anterior cingulate and orbitofrontal cortex increased among the excessive Internet game playing group following exposure to Internet video game cues relative to general players. They also reported that increased craving for Internet video games correlated with increased activity in the anterior cingulate for all participants. This quasi-experimental study is insightful for it not only offered evidence for a dissimilar brain activity in online gaming addicts compared to a general player control group, but it also elucidated brain activation that occurs as a consequence of playing in both groups. This indicates that (i) craving for online games alters brain activity irrespective of addiction status and might therefore be seen as a (prodromal) symptom of addiction, and that (ii) addicted players can be distinguished from non-addicted online gamers by a different form of brain activation.

Liu *et al*. [45] administered the regional homogeneity (ReHo) method to analyze encephalic functional characteristics of Internet addicts under resting state. The sample comprised 19 college students with Internet addiction and 19 controls. Internet addiction was assessed using Beard and Wolf’s criteria [72]. FMRI using 3.0T Siemens Tesla Trio Tim scanner was performed. Regional homogeneity indicates temporal homogeneity of brain oxygen levels in brain regions of interest. It was reported that Internet addicts suffered from functional brain changes leading to abnormalities in regional homogeneity relative to the control group, particularly concerning the reward pathways traditionally associated with substance addictions. Among Internet addicts, brain regions in ReHo in resting state were increased (cerebellum, brainstem, rCG, bilateral parahippocampus (blPHipp), right frontal lobe, left superior frontal gyrus (lSFG), right inferior temporal gyrus (rITG), left superior temporal gyrus (lSTG) and middle temporal gyrus (mTG)), relative to the control group. The temporal regions are involved in auditory processing, comprehension and verbal memory, whereas the occipital regions take care of visual processing. The cerebellum regulates cognitive activity. The cingulate gyrus pertains to integrating sensory information, and monitoring conflict. The hippocampi are involved in the brain’s mesocorticolimbic system that is associated with reward pathways. Taken together, these findings provide evidence for a change in a variety of brain regions as a consequence of Internet addiction. As this study assessed regional homogeneity under a resting state, it is unclear whether the changes in the brain observed in Internet addicts are a cause or consequence of the addiction. Therefore, no causal inferences can be drawn. 

Yuan *et al*. [46] investigated the effects of Internet addiction on the microstructural integrity of major neuronal fiber pathways and microstructural changes associated with the duration of Internet addiction. Their sample comprised 18 students with Internet addiction (12 males; mean age = 19.4, SD = 3.1 years; mean online gaming = 10.2 h per day, SD = 2.6; duration of Internet addiction = 34.8 months, SD = 8.5), and 18 non-Internet addicted control participants (mean age = 19.5 years, SD = 2.8). All participants completed the Modified Diagnostic Questionnaire for Internet Addiction [72], a Self-Rating Anxiety Scale (no details provided), and a Self-Rating Depression Scale (no details provided). The authors employed fMRI and used the optimized voxel-based morphometry (VBM) technique. They analyzed white matter fractional anisotropy (FA) changes by using diffusion tensor imaging (DTI) to discern brain structural changes as a consequence of Internet addiction length. The results showed that Internet addiction resulted in changes in brain structure, and that the brain changes found appear similar to those found in substance addicts. 

Controlling for age, gender, and brain volume, it was found that among Internet addicts there was decreased gray matter volume in the bilateral dorsolateral prefrontal cortex (DLPFC), supplementary motor area (SMA), orbitofrontal cortex (OFC), cerebellum and the left rostral ACC (rACC), an increased FA of the left posterior limb of the internal capsule (PLIC), and reduced FA in white matter in the right parahippocampal gyrus (PHG). There was also a correlation between gray matter volumes in DLPFC, rACC, SMA, and white matter FA changes of PLIC with the length of time the person had been addicted to the Internet. This indicates that the longer a person is addicted to the Internet, the more severe brain atrophy becomes. In light of the method, it is unclear from the authors’ description in how far their sample included those who were addicted to the Internet *per se*, or to playing games online. The inclusion of a specific question asking about the frequency and duration of online gaming (rather than any potential other Internet activity) suggests that the group in question consisted of gamers. In addition to this, the presented findings cannot exclude any other factor that may be associated with Internet addiction (e.g., depressive symptomatology) that may have contributed to the increased severity of brain atrophy. 

Dong *et al*. [39] examined reward and punishment processing in Internet addicts compared to healthy controls. Adult males (*n* = 14) with Internet addiction (mean age = 23.4, SD = 3.3 years) were compared to 13 healthy adult males (mean age = 24.1 years, SD = 3.2). Participants completed a structured psychiatric interview [79], the Beck Depression Inventory [78], the Chinese Internet Addiction Test [62,63], and the Internet Addiction Test (IAT; [61]). The IAT measures psychological dependence, compulsive use, withdrawal, related problems in school, work, sleep, family, and time management. Participants had to score over 80 (out of 100) on the IAT to be classed as having Internet addiction. Furthermore, all those classed as Internet addicts spent more than six hours online every day (excluding work-related Internet use) and had done so for a period of more than three months. 

All the participants engaged in a reality-simulated guessing task for money gain or loss situation using playing cards. The participants underwent fMRI with stimuli presented through a monitor in the head coil, and their blood oxygen level dependence (BOLD) activation was measured in relation to wins and losses on the task. The results showed that Internet addiction was associated with increased activation in the OFC in gain trials, and decreased anterior cingulate activation in loss trials compared to normal controls. Internet addicts showed enhanced reward sensitivity and decreased loss sensitivity when compared with the control group [39]. The quasi-experimental nature of this study allowed for an actual comparison of the two groups by exposing them to a gaming situation and thus artificially inducing a neuronal reaction that was a consequence of the engagement in the task. Therefore, this study allowed for the extrication of a causal relationship between exposure to gaming cues and the resulting brain activation. This may be considered as empirical proof for reward sensitivity in Internet addicts relative to healthy controls.

Han *et al*. [40] compared regional gray matter volumes in patients with online gaming addiction and professional gamers. The authors carried out fMRI using a 1.5 Tesla Espree scanner (Siemens, Erlangen) and carried out a voxel-wise comparison of gray matter volume. All participants completed the Structured Clinical Interview for DSM-IV [80], the Beck Depression Inventory [78], the Barratt Impulsiveness Scale-Korean version (BIS-K9) [81,82], and the Internet Addiction Scale (IAS) [67]. Those (i) scoring over 50 (out of 100) on the IAS, (ii) playing for more than four hours per day/30 h per week, and (iii) impaired behavior or distress as a consequence of online game play were classed as Internet gaming addicts. The sample comprised three groups. The first group included 20 patients with online gaming addiction (mean age = 20.9, SD = 2.0; mean illness duration = 4.9 years, SD = 0.9; mean playing time = 9.0, SD = 3.7 h/day; mean Internet use = 13.1, SD = 2.9 h/day; mean IAS scores = 81.2, SD = 9.8). The second group was comprised of 17 professional gamers (mean age = 20.8 years, SD = 1.5; mean playing time = 9.4, SD = 1.6 h/day; mean Internet use = 11.6, SD = 2.1 h/day; mean IAS score = 40.8, SD = 15.4). The third group included 18 healthy controls (mean age = 12.1, SD = 1.1 years; mean gaming = 1.0, SD = 0.7 h/day; mean Internet use = 2.8, SD = 1.1 h/day; mean IAS score = 41.6, SD = 10.6). 

The results showed that gaming addicts had higher impulsiveness, perseverative errors, increased volume in left thalamus gray matter, and decreased gray matter volume in ITG, right middle occipital gyrus (rmOG), and left inferior occipital gyrus (lIOG) relative to the control group. Professional gamers had increased gray matter volume in lCG, and decreased gray matter in lmOG and rITG relative to the control group, increased gray matter in lCG, and decreased left thalamus gray matter relative to the problem online gamers. The main differences between the gaming addicts and the professional gamers lay in the professional gamers’ increased gray matter volumes in lCG (important for executive function, salience, and visuospatial attention) and gaming addicts’ left thalamus (important in reinforcement and alerting) [40]. Based on the non-experimental nature of the study, it is difficult to attribute the evinced dissimilarities in brain structure across groups to the actual addiction status. Possible confounding variables cannot be excluded that may have contributed to the differences found.

Han *et al*. [41] tested the effects of bupropion sustained release treatment on brain activity among Internet gaming addicts and healthy controls. All participants completed the Structured Clinical Interview for DSM-IV [80], the Beck Depression Inventory [78], the Internet Addiction Scale [61], and the Craving for Internet video game play was assessed with a 7-point visual analogue scale. Those participants who engaged in Internet gaming for more than four hours a day, scored more than 50 (out of 100) on the IAS, and had impaired behaviors and/or distress were classed as Internet gaming addicts. The sample comprised 11 Internet gaming addicts (mean age = 21.5, SD = 5.6 years; mean craving score = 5.5, SD = 1.0; mean playing time = 6.5, SD = 2.5 h/day; mean IAS score = 71.2, SD = 9.4), and 8 healthy controls (mean age = 11.8, SD = 2.1 years; mean craving score = 3.9, SD =1.1; mean Internet use = 1.9, SD = 0.6 h/day; mean IAS score = 27.1, SD = 5.3). During exposure to game cues, Internet gaming addicts had more brain activation in left occipital lobe cuneus, left dorsolateral prefrontal cortex, and left parahippocampal gyrus relative to the control group. Participants with Internet gaming addiction underwent six weeks of bupropion sustained release treatment (150 mg/day for first week, and 300 mg/day afterwards). Brain activity was measured at baseline and after treatment using a 1.5 Tesla Espree fMRI scanner. The authors reported that bupropion sustained release treatment works for Internet gaming addicts in a similar way as it works for patients with substance dependence. After treatment, craving, play time, and cue-induced brain activity decreased among Internet gaming addicts. The longitudinal nature of this study allows for a determination of cause and effect, which emphasizes the validity and reliability of the presented findings.

### 3.2. sMRI Studies

Lin *et al*. [48] investigated white matter integrity in adolescents with Internet addiction. All participants completed a modified version of the Internet Addiction Test [72], the Edinburgh handedness inventory [83], the Mini International Neuropsychiatric Interview for Children and Adolescents (MINI-KID) [84], the Time Management Disposition Scale [85], the Barratt Impulsiveness Scale [86], the Screen for Child Anxiety Related Emotional Disorders (SCARED) [87], and the Family Assessment Device (FAD) [88]. The sample comprised 17 Internet addicts (14 males; age range = 14–24 years; IAS mean score = 37.0, SD = 10.6), and 16 healthy controls (14 males; age range = 16–24 years; IAS mean score = 64.7, SD = 12.6). The authors carried out a whole brain voxel-wise analysis of fractional anisotropy (FA) by tract-based spatial statistics (TBSS), and volume of interest analysis was performed using diffusion tensor imaging (DTI) via a 3.0-Tesla Phillips Achieva medical scanner. 

The results indicated that the OFC was associated with emotional processing and addiction-related phenomena (e.g., craving, compulsive behaviors, maladaptive decision-making). Abnormal white matter integrity in the anterior cingulate cortex was linked to different addictions, and indicated an impairment in cognitive control. The authors also reported impaired fiber connectivity in the corpus callosum that is commonly found in those with substance dependence. Internet addicts showed lower FA throughout the brain (orbito-frontal white matter corpus callosum, cingulum, inferior fronto-occipital fasciculus, corona radiation, internal and external capsules) relative to controls, and there were negative correlations between FA in the left genu of corpus callosum and emotional disorders, and FA in the left external capsule and Internet addiction. Overall, Internet addicts had abnormal white matter integrity in brain regions linked to emotional processing, executive attention, decision-making and cognitive control compared to the control group. The authors highlighted similarities in brain structures between Internet addicts and substance addicts [48]. Given the non-experimental and cross-sectional nature of the study, alternative explanations for brain alterations other than addiction cannot be excluded.

Zhou *et al*. [47] investigated brain gray matter density (GMD) changes in adolescents with Internet addiction using voxel-based morphometry (VBM) analysis on high-resolution T1-weighted structural magnetic resonance images. Their sample comprised 18 adolescents with Internet addiction (16 males; mean age = 17.2 years, SD = 2.6), and 15 healthy control participants with no history of psychiatric illness (13 males; mean age = 17.8 years, SD = 2.6). All participants completed the modified Internet Addiction Test [72]. The authors used high-resolution T1-weighted MRIs performed on a 3T MR scanner (3T Achieva Philips), scanned MPRAGE pulse sequences for gray and white matter contrasts, and VBM analysis was used to compare GMD between groups. Results showed that Internet addicts had lower GMD in the lACC (necessary for motor control, cognition, motivation), lPCC (self-reference), left insula (specifically related to craving and motivation), and the left lingual gyrus (*i.e.*, areas that are linked to emotional behavior regulation and thus linked to emotional problems of Internet addicts). The authors state that their study provided neurobiological proof for structural brain changes in adolescents with Internet addiction, and that their findings have implications for the development of addiction psychopathology. Despite the differences found between the groups, the findings cannot exclusively be attributed to the addiction status of one of the groups. Possible confounding variables may have had an influence on brain changes. Moreover, the directionality of the relationship cannot be explained with certainty in this case.

### 3.3. EEG Studies

Dong *et al*. [53] investigated response inhibition among Internet addicts neurologically. The recordings of event-related brain potentials (ERPs) via EEG were examined in 12 male Internet addicts (mean age = 20.5 years, SD = 4.1) and compared with 12 healthy control university students (mean age = 20.2, SD = 4.5) while undergoing a go/NoGo task. The participants completed psychological tests (*i.e.*, Symptom Checklist-90 and 16 Personal Factors scale [89]) and the Internet Addiction Test [65]. The results showed that Internet addicts had lower NoGo-N2 amplitudes (representing response inhibition—conflict monitoring), higher NoGo-P3 amplitudes (inhibitory processes—response evaluation), and longer NoGo-P3 peak latency when compared to controls. The authors concluded that compared to the control group, Internet addicts (i) had lower activation in conflict detection stage, (ii) used more cognitive resources to complete the later stage of the inhibition task, (iii) were less efficient at information processing, and (iv) had lower impulse control. 

Dong *et al*. [52] compared Internet addicts and healthy controls on event-related potentials (ERP) via EEG while they were performing a color-word Stroop task. Male participants (*n* = 17; mean age = 21.1 years, SD = 3.1) and 17 male healthy university students (mean age = 20.8 years, SD = 3.5) completed psychological tests (*i.e.*, the Symptom Checklist-90 and the 16 Personal Factors scale [89]) and the Internet Addiction Test [64]. This version of the IAT included eight items (preoccupation, tolerance, unsuccessful abstinence, withdrawal, loss of control, interests, deception, escapism motivation) and the items were scored dichotomously. Those participants who endorsed four or more items were classed as Internet addicts. Results showed that Internet addicts had a longer reaction time and more response errors in incongruent conditions compared to controls. The authors also reported reduced medial frontal negativity (MFN) deflection in incongruent conditions than controls. Their findings suggested that Internet addicts have impaired executive control ability compared to controls.

Ge *et al*. [55] investigated the association between the P300 component and Internet addiction disorder among 86 participants. Of these, 38 were Internet addiction patients (21 males; mean age = 32.5, SD = 3.2 years) and 48 were healthy college student controls (25 males; mean age = 31.3, SD = 10.5 years). In an EEG study, P300 ERP was measured using a standard auditory oddball task using the American Nicolet BRAVO instrument. All participants completed the Structured Clinical Diagnostic Interview for Mental Disorders [80], and the Internet Addiction Test [64]. Those who endorsed five or more (of the eight items) were classed as Internet addicts. The study found that Internet addicts had longer P300 latencies relative to the control group, and that Internet addicts had similar profiles as compared to other substance-related addicts (*i.e.*, alcohol, opioid, cocaine) in similar studies. However, the results did not indicate that Internet addicts had a deficiency in perception speed and auditory stimuli processing. This appears to indicate that rather than being detrimental to perception speed and auditory stimuli processing, Internet addiction may have no effect on these specific brain functions. The authors also reported that the cognitive dysfunctions associated with Internet addiction can be improved via cognitive-behavioral therapy and that those who participated in cognitive-behavioral therapy for three months decreased their P300 latencies. The final longitudinal result is particularly insightful because it assessed the development over time that may be attributed to the beneficial effects of therapy.

Little *et al*. [56] investigated error-processing and response inhibition in excessive gamers. All participants completed the Videogame Addiction Test (VAT) [73], the Dutch version of the Eysenck Impulsiveness Questionnaire [90,91], and the Quantity-Frequency-Variability Index for alcohol consumption [92]. The sample comprised 52 students grouped into two groups of 25 excessive gamers (23 males; scoring more than 2.5 on VAT; mean age = 20.5, SD = 3.0 years; mean VAT score = 3.1, SD = 0.4; average gaming = 4.7 h a day, SD = 2.3) and 27 controls (10 males; mean age = 21.4, SD = 2.6; mean Vat score = 1.1, SD = 0.2; average gaming = 0.5 h a day, SD = 1.2). The authors used a Go/NoGo paradigm using EEG and ERP recordings. Their findings indicated similarities with substance dependence and impulse control disorders in relation to poor inhibition and high impulsivity in excessive gamers relative to the control group. They also reported that excessive gamers had reduced fronto-central ERN amplitudes following incorrect trials in comparison to correct trials and that this led to poor error-processing. Excessive gamers also displayed less inhibition on both self-report and behavioral measures. The strength of this study include its quasi-experimental nature as well as the verification of self-reports with behavioral data. Therefore, validity and reliability of the findings are increased. 

### 3.4. SPECT Studies

Hou *et al*. [51] examined reward circuitry dopamine transporter levels in Internet addicts compared to a control group. The Internet addicts comprised five males (mean age = 20.4, SD = 2.3) whose mean daily Internet use was 10.2 h (SD = 1.5) and who had suffered from Internet addiction for more than six years. The age-matched control group comprised nine males (mean age = 20.4, SD = 1.1 years), whose mean daily use was 3.8 h (SD = 0.8 h). The authors performed 99mTc-TRODAT-1 single photon emission computed tomography (SPECT) brain scans using Siemens Diacam/e.cam/icon double detector SPECT. They reported that reduced dopamine transporters indicated addiction and that there were similar neurobiological abnormalities with other behavioral addictions. They also reported that striatal dopamine transporter (DAT) levels decreased among Internet addicts (necessary for regulation of striatal dopamine levels) and that volume, weight, and uptake ratio of the corpus striatum were reduced relative to controls. Dopamine levels were reported to be similar to people with substance addictions and that Internet addiction “may cause serious damages to the brain” ([51], p. 1). This conclusion cannot be seen as entirely accurate for the directionality of the reported effect cannot be established with the utilized method.

### 3.5. PET Studies

Koepp *et al*. [50] were the first research team to provide evidence for striatal dopamine release during video game play (*i.e.*, a game navigating a tank for monetary incentive). In their study, eight male video game players (age range = 36–46 years) underwent positron emission tomography (PET) during video game play and under resting condition. The PET scans employed a 953B-Siemens/CTIPET camera, and a region-of-interest (ROI) analysis was performed. Extracellular dopamine levels were measured via differences in [^11^C]RAC-binding potential to dopamine D_2_ receptors in ventral and dorsal striata. The results showed that ventral and dorsal striata were associated with goal-directed behavior. The authors also reported that the change of binding potential during video game play was similar to that following amphetamine or methylphenidate injections. In light of this, the earliest study included in this review [50] was already able to highlight changes in neurochemical activity as a consequence of gaming relative to a resting control. This finding is of immense significance because it clearly indicates that the activity of gaming can in fact be compared to using psychoactive substances when viewed from a biochemical level.

Kim *et al*. [49] tested whether Internet addiction was associated with reduced levels of dopaminergic receptor availability in the striatum. All participants completed the Structured Clinical Interview for DSM-IV [80], the Beck Depression Inventory [93], the Korean Wechsler Adult Intelligence Scale [94], the Internet Addiction Test [69] and the Internet Addictive Disorder Diagnostic Criteria (IADDC; [68]). Internet addiction was defined as those participants who scored more than 50 (out of 100) on the IAT, and endorsed three or more of the seven criteria on the IADDC. 

Their sample comprised five male Internet addicts (mean age = 22.6, SD = 1.2 years; IAT mean score = 68.2, SD = 3.7; mean daily Internet hours = 7.8, SD = 1.5) and seven male controls (mean age = 23.1, SD = 0.7 years; IAT mean score = 32.9, SD = 5.3; mean daily Internet hours = 2.1, SD = 0.5). The authors carried out a PET study and used a radiolabeled ligand [^11^C]raclopride and positron emission tomography via ECAT EXACT scanner to test dopamine D_2_ receptor binding potential. They also performed fMRI using a General Electric Signa version 1.5T MRI scanner. The method for assessing D_2_ receptor availability examined regions of interest (ROI) analysis in ventral striatum, dorsal caudate, dorsal putamen. The authors reported that Internet addiction was found to be related to neurobiological abnormalities in the dopaminergic system as found in substance-related addictions. It was also reported that Internet addicts had reduced dopamine D_2_ receptor availability in the striatum (*i.e.*, bilateral dorsal caudate, right putamen) relative to the controls, and that there was a negative correlation of dopamine receptor availability with Internet addiction severity [49]. However, from this study it is unclear to what extent Internet addiction may have caused the differences in neurochemistry relative to any other confounding variable, and, similarly, whether it is the different neurochemistry that may have led to the pathogenesis. 

## 4. Discussion

The results of the fMRI studies indicate that brain regions associated with reward, addiction, craving, and emotion are increasingly activated during game play and presentation of game cues, particularly for addicted Internet users and gamers, including the NAc, AMG, AC, DLPFC, IC, rCN, rOFC, insula, PMC, precuneus [42,43]. Gaming cues appeared as strong predictors of craving in male online gaming addicts [44]. Moreover, it was shown that associated symptoms, such as craving, gaming cue-induced brain activity, and cognitive dysfunctions can be reduced following psychopharmacological or cognitive-behavioral treatment [41,55].

In addition to this, structural changes have been demonstrated in Internet addicts relative to controls, including the cerebellum, brainstem, rCG, blPHipp, right frontal lobe, lSFG, rITG, lSTG, and mTG. Specifically, these regions appeared to be increased and calibrated, indicating that in Internet addicts, neuroadaptation occurs that synchronizes a variety of brain regions. These include, but are not limited to, the widely reported mesocorticolimbic system involved in reward and addiction. In addition, Internet addicts’ brains appear to be able to integrate sensorimotor and perceptual information better [45]. This may be explained by a frequent engagement with Internet applications such as games, which require a stronger connectivity between brain regions in order for learned behaviors and reactions to addiction-relevant cues to occur automatically.

Furthermore, compared to controls, Internet addicts were found to have decreased gray matter volume in the blDLPFC, SMA, OFC, cerebellum, ACC, lPCC, increased FA lPLIC, and decreased FA in white matter in the PHG [46]. The lACC is necessary for motor control, cognition, and motivation, and its decreased activation has been linked to cocaine addiction [95]. The OFC is involved in processing emotions and it plays a role in craving, maladaptive decision-making processes, as well as the engagement in compulsive behaviors, each of which are integral to addiction [96]. Moreover, the length of Internet addiction correlated with changes in DLPFC, rACC, SMA, and PLIC, testifying to the increase of brain atrophy severity over time [46]. The DLPFC, rACC, ACC, and PHG have been linked to self-control [22,25,44], whereas the SMA mediates cognitive control [97]. Atrophy in these regions can explain the loss of control an addict experiences in regards to his drug or activity of choice. The PCC, on the other hand, is important in mediating emotional processes and memory [98], and a decrease in its gray matter density may be indicative of abnormalities associated with these functions.

The increase of the internal capsule has been linked to motor hand function and motor imagery [99,100], and can possibly be explained by the frequent engagement in computer games, that requires and significantly improves eye-hand coordination [101]. Moreover, decreased fiber density and white matter myelination as measured with FA were found in the anterior limb of the internal capsule, external capsule, corona radiation, inferior fronto-occipital fasciculus and precentral gyrus in Internet addicts relative to healthy controls [48]. Similar white matter abnormalities have been reported in other substance-related addictions [102,103]. Similarly, fiber connectivity in the corpus callosum was found to be decreased in Internet addicts relative to healthy controls, which indicates that Internet addiction may have similar degenerative consequences with regards to links between the hemispheres. These findings are in accordance with those reported in substance-related addictions [104]. 

Moreover, there appeared gender differences in activation in such a way that for males, the activation and connectivity of brain regions associated with the mesocorticolimbic reward system were stronger relative to females. This may explain the significantly higher vulnerability for males to develop an addiction to gaming and the Internet that has been reported in reviews of the empirical literature (*i.e.*, [7,105]).

In addition to the MRI findings, the EEG studies assessing Internet and gaming addiction to date offer a variety of important findings that may help in understanding behavioral and functional correlates of this emergent psychopathology. In addition to this, the experimental nature of all of the included EEG studies allows for the determination of a causal relationship between the assessed variables. It has been shown that compared to controls, Internet addicts had decreased P300 amplitudes and an increased P300 latency. Typically, this amplitude reflects attention allocation. The differences in amplitude between Internet addicts and controls indicate that either Internet addicts have an impaired capacity for attention or they are not able to allocate attention adequately [55,57]. Small P300 amplitudes have been associated with genetic vulnerability for alcoholism in a meta-analysis [106]. Decreased P300 latency furthermore was found to distinguish heavy social drinkers from low social drinkers [107]. Accordingly, there appears to be a common change in neuronal voltage fluctuations in persons addicted to substances and the engagement in Internet use relative to people who are not addicted. Accordingly, Internet addiction appears to have an effect on neuroelectric functioning that is similar to substance addictions. Generally, Internet addicts’ brains appeared to be less efficient with regards to information processing and response inhibition relative to healthy control participants’ brains [54,56]. This indicates that Internet addiction is associated with low impulse control, and the use of an increased amount of cognitive resources in order to complete specific tasks [53]. Furthermore, Internet addicts appear to have an impaired executive control ability relative to controls [56,53]. These results are in accordance with reduced executive control ability found in cocaine addicts, implicating decreased activity in pre- and midfrontal brain regions that would allow for impulse-driven actions [108]. 

From a biochemical point of view, the results of PET studies provide evidence for striatal dopamine release during gaming [50]. Frequent gaming and Internet use were shown to decrease dopamine levels (due to decreased dopamine transporter availability) and lead to neurobiological dysfunctions in the dopaminergic system in Internet addicts [49,51]. The decreased availability was linked with the severity of Internet addiction [49]. Reduced dopamine levels have been reported in addictions time and again [26,109,110]. Furthermore, structural abnormalities of the corpus striatum have been reported [51]. Damages to the corpus striatum have been associated with heroin addiction [111].

The studies included in this literature review appear to provide compelling evidence for the similarities between different types of addictions, notably substance-related addictions and Internet addiction, on a variety of levels. On the molecular level, it has been shown that Internet addiction is characterized by an overall reward deficiency that is characterized by decreased dopaminergic activity. The direction of this relationship is yet to be explored. Most studies could not exclude that an addiction develops as a consequence of a deficient reward system rather than vice versa. The possibility that deficits in the reward system predispose certain individuals to develop a drug or a behavioral addiction such as Internet addiction may put an individual at greater risk for psychopathology. In Internet addicts, negative affectivity can be considered the baseline state, where the addict is preoccupied with using the Internet and gaming to modify his mood. This is brought about by the activation of the antireward system. Due to the excessive use of the Internet and online gaming, opponent processes appear to be set in motion that quickly habituate the addict to the engagement with the Internet, leading to tolerance, and, if use is discontinued, withdrawal [27]. Accordingly, decreased neuronal dopamine as evinced in Internet addiction may be linked to commonly reported comorbidities with affective disorders, such as depression [112], bipolar disorder [113], and borderline personality disorder [10].

On the level of neural circuitry, neuroadaptation occurs as a consequence of increased brain activity in brain areas associated with addiction and structural changes as a consequence of Internet and gaming addiction. The cited studies provide a clear picture of Internet and gaming addiction pathogenesis and stress how maladaptive behavioral patterns indicative of addiction are maintained. The brain adapts to frequent use of drugs or engagement in addictive behaviors so that it becomes desensitized to natural reinforcers. Importantly, functioning and structure of the OFC and cingulate gyrus are altered, leading to increased drug or behavior salience and loss of control over behaviors. Learning mechanisms and increased motivation for consumption/engagement result in compulsive behaviors [114].

On a behavioral level, Internet and gaming addicts appear to be constricted with regards to their impulse control, behavioral inhibition, executive functioning control, attentional capabilities, and overall cognitive functioning. In turn, certain skills are developed and improved as a consequence of frequent engagement with the technology, such as the integration of perceptual information into the brain via the senses, and hand-eye coordination. It appears that the excessive engagement with the technology results in a number of advantages for players and Internet users, however to the detriment of fundamental cognitive functioning. 

Taken together, the research presented in this review substantiates a *syndrome model of addictions* for there appear to be neurobiological commonalities in different addictions [115]. According to this model, neurobiology and psychosocial context increase the risk to become addicted. The exposure to the addictive drug or behavior and specific negative events and/or the continued use of the substance and engagement in the behavior leads to behavioral modification. The consequence is the development of full-blown addictions, that are different in expression (e.g., cocaine, the Internet and gaming), but similar in symptomatology [115], *i.e.*, mood modification, salience, tolerance, withdrawal, conflict, and relapse [6]. 

Notwithstanding the insightful results reported, a number of limitations need to be addressed. First, there appear methodological problems that may decrease the strength of the reported empirical findings. The reported brain changes associated with Internet and online gaming addiction described in this review may be explained in two different ways. On the one hand, one could argue that Internet addiction leads to brain alterations relative to controls. On the other hand, people with unusual brain structures (as the ones observed in the present study) may be particularly predisposed to developing addictive behaviors. Only experimental studies will allow a determination of cause and effect relationships. Given the sensitive nature of this research that essentially assesses potential psychopathology, ethical considerations will limit the possibilities of experimental research in the field. In order to overcome this problem, future researchers should assess brain activity and brain alterations on a number of occasions during a person’s life longitudinally. This would allow for the extrication of invaluable information with regards to the relationships of pathogenesis and related brain changes in a more elaborate and, importantly, causal fashion.

Secondly, this review included neuroimaging studies of both Internet addicts and online gaming addicts. Based on the collected evidence, it appears difficult to make any deductions as regards the specific activities the addicts engaged in online, other than some authors specifically addressing online gaming addiction. Others, on the other hand, used the categories Internet addiction and Internet gaming addiction almost interchangeably, which does not allow for any conclusions with regards to differences and similarities between the two. In light of this, researchers are advised to clearly assess the actual behaviors engaged in online, and, if appropriate, extend the notion of gaming to other potentially problematic online behaviors. Ultimately, people do not become addicted to the medium of the Internet *per sé,* but it is rather the activities that they engage in that may be potentially problematic and could lead to addictive online behavior.

## 5. Conclusions

This review aimed to identify all empirical studies to date that have used neuroimaging techniques in order to discern the neuronal correlates of Internet and gaming addiction. There are relatively few studies (*n* = 19), and therefore it is crucial to conduct additional studies to replicate the findings of those already carried out. The studies to date have used both structural and functional paradigms. The use of each of these paradigms allows for the extrication of information that is crucial for establishing altered neuronal activity and morphology as precipitated by Internet and gaming addiction. Overall, the studies indicate that Internet and gaming addiction is associated with both changes in function as well as structure of the brain. Therefore, not only does this behavioral addiction increase the activity in brain regions commonly associated with substance-related addictions, but it appears to lead to neuroadaptation in such a way that the brain itself actually changes as a consequence of excessive engagement with the Internet and gaming.

In terms of the method, neuroimaging studies offer an advantage over traditional survey and behavioral research because, using these techniques, it is possible to distinguish particular brain areas that are involved in the development and maintenance of addiction. Measurements of increased glutamatergic and electrical activity give insight into brain functioning, whereas measures of brain morphometry and water diffusion provide an indication of brain structure. It has been shown that each of these undergoes significant changes as a consequence of Internet and gaming addiction.

To conclude, understanding the neuronal correlates associated with the development of addictive behaviors related to using the Internet and playing online games will promote future research and will pave the way for the development of addiction treatment approaches. In terms of clinical practice, increasing our knowledge regarding the pathogenesis and maintenance of Internet and gaming addiction is essential for the development of specific and effective treatments. These include psychopharmacological approaches that target Internet and gaming addiction specifically on the level of biochemistry and neurocircuitry, as well as psychological strategies, that aim to modify learned maladaptive cognitive and behavioral patterns.
